# Procedure of Eliminating Porosity in Grey Cast Iron with Low Sulphur Content

**DOI:** 10.3390/ma15186273

**Published:** 2022-09-09

**Authors:** Józef Dorula, Dariusz Kopyciński, Edward Guzik, Andrzej Szczęsny

**Affiliations:** 1 Faculty of Foundry Engineering, AGH University of Science and Technology, al. A. Mickiewicza 30, 30-059 Kraków, Poland; 2 Vesuvius Poland, Foseco Foundry Operation in Gliwice, St. Leonarda da Vinci 5, 44-109 Gliwice, Poland

**Keywords:** grey cast iron, porosity, inoculation, ITACA thermal analysis

## Abstract

This study shows that the inoculation process of a molten alloy is crucial in disposing of porosity-type defects. A thermal analysis is used to assess the physico-chemical state of a molten alloy, which can be an indicator of the inoculation effect. A modern thermal analysis should be able to perform a quick data-analysis and provide information about any possible problems in a casting if it is poured with the analysed alloy. The time of the transmission of this information depends on whether we can make a decision and introduce changes to the metallurgical process. An important piece of information that can be obtained in this way is a message about the possibility of the appearance of porosity in a cast iron casting. In such a situation, an operator can react by applying an additional dose of inoculant. The porosity that is indicated by the thermal analysis systems can be either gaseous or shrinkage in nature. The research that is presented in this paper is based on two industrial castings that are made of cast iron with reduced sulphur content, in which shrinkage porosity occurred and was detected during the mechanical machining of the castings. As a result of laboratory tests in which iron powder was introduced along with an inoculant, a mixture was developed that, when applied under industrial conditions, eliminated the porosity defects by increasing the number of austenite dendrites. The ITACA thermal analysis system was used at each stage of the research, which allowed for the faster and more precise determination of the appropriate amount of the inoculant mixture that was used.

## 1. Introduction

**Figure 1:**
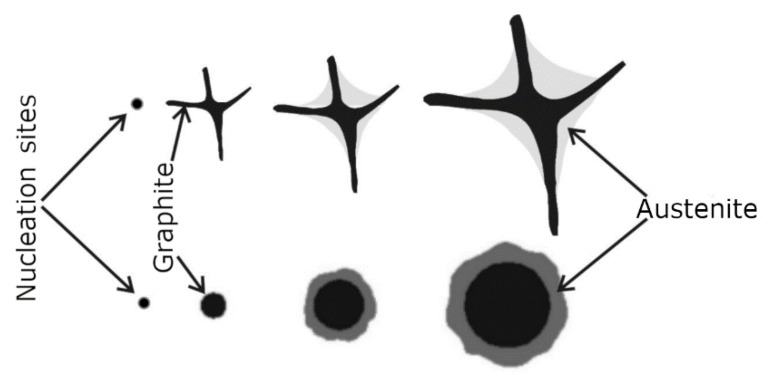
Sequence of shaping in grey cast iron of eutectic grains with flake and nodular graphite.

**Figure 2:**
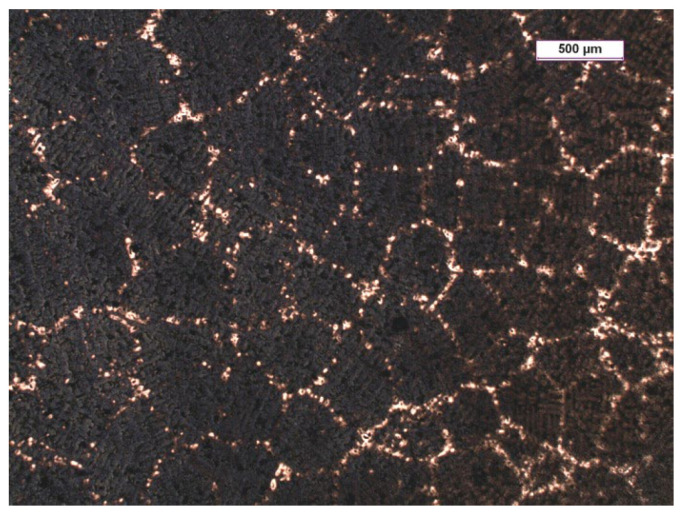
Example of grey cast iron microstructure consisting of eutectic grains revealed on surface of metallographic specimen after etching with Stead’s solution.

Grey cast iron is a casting alloy in which a graphite eutectic is produced by its structure during crystallisation. This occurs when the molten alloy in a casting mould begins to crystallise within the temperature range that is between the equilibrium temperature of the eutectic transition for the stable Tst and the metastable Tmst of the Fe-C phase equilibrium system. In this case, the molten alloy will be supercooled with respect to the liquidus temperature for the austenite and graphite; therefore, the eutectic graphite crystallises directly from the liquid. On the other hand, the graphite nucleus is initially shaped as a pre-eutectic phase; depending on the growth conditions, it takes the shape of a flake or a nodule (spherical) (which is shown schematically in Figure 1). A compact (vermicular) form of graphite may also arise as an intermediate between the flakes and spherical shapes. Next, austenite grows on the graphite surface; after the completion of the crystallisation, a eutectic grain forms with the graphite. In grey cast iron with flake graphite, the eutectic grains have a continuous graphite skeleton that is filled with austenite. The appearance of the surface of the metallographic specimen of the grey cast iron sample on which the eutectic grains were revealed on a macro scale is shown in Figure 2. It is worth noting that the area of the cast iron microstructure that can be observed on the surface of the metallographic specimen corresponds only to the accidental intersection of the plane with the graphite grain skeleton [1].

Using the example of hypoeutectic grey cast iron, it is possible to illustrate the influence of the primary grains of austenite dendrites (which crystallise some time in advance) on the formation of the eutectic grains, as well as on the type of microstructure that is present in the cast iron. When the growth of the eutectic grains is blocked by the austenite dendrites, the interfacial distance of the eutectic is determined by the interdendritic distance. Depending on the mutual positions of the eutectic grains, primary austenite, and their shares in the casting volume, four basic types of distribution of flake graphite in grey cast iron’s microstructure can be identified (described by the ASTM A 247-47 and PN-EN ISO 945 standards). The [2] presents the probable sequence of the formation of a micro-structure of Types A, B, D, and E in hypoeutectic grey cast iron. It is generally known that the different types of graphite distribution have a significant impact on the mechanical properties of the cast iron. Generally, the aim is to obtain an A-type structure in a grey cast iron structure (graphite that is evenly distributed in the matrix). In industrial practice, this is achievable only with a good inoculation treatment that affects the eutectic grains of the graphite. In addition, the mechanical properties of the casting will also have this macrostructure, i.e., the number of primary austenite grains. These grains also influence whether there will be defects in a porosity-type casting.

**Figure 3:**
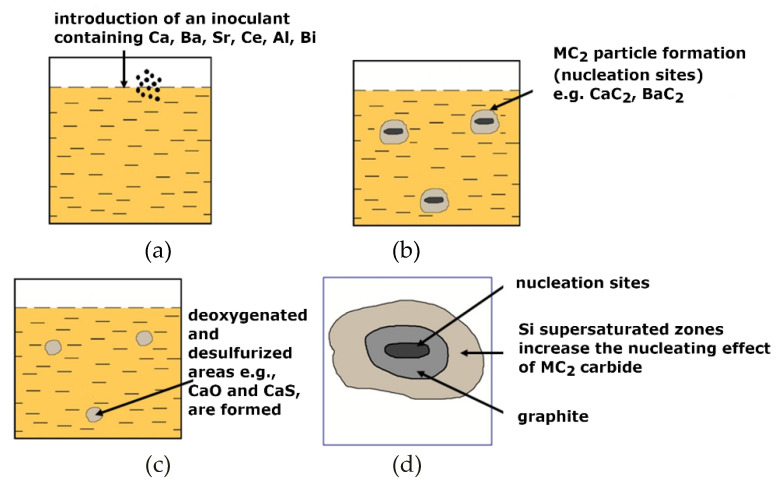
(**a**) Scheme of procedure for inoculation of grey cast iron: introduction of inoculant containing Ca, Ba, Sr, and Al; (**b**) formation of deoxidised and desulphurised areas (e.g., CaO and CaS); (**c**) formation of MC_2_ (CaC_2_, BaC_2_) particles (nucleation sites) on which graphite nucleates; (**d**) schematic of inoculant interaction in molten metal.

According to the authors of this paper, the most probable inoculation hypothesis is the theory of B. Lux [3,4] in which carbides that are formed in molten metal are presented as nucleation sites of graphite. The instability of carbides under the influence of the oxygen and sulphur that are contained in molten metal [5,6,7] is the basis of this theory. The scheme of the cast iron inoculation procedure can be presented as shown in Figure 3.

Undoubtedly, the most important process-evaluation indicator is an increase in the number of eutectic grains. Moreover, the changes in the characteristics of the graphite particles are assessed after the inoculation procedure. In the structure of the inoculated cast iron, the flake graphite is evenly distributed (Type A) at the expense of the graphite with an interdendritic arrangement of Type D (which occurred in the reference cast iron). Moreover, the value of the degree of supercooling (ΔT) and the tendency of cast iron to whiten during the crystallisation of the graphite eutectic decreases. The microstructure of the inoculated cast iron is dominated by a pearlitic metal matrix. It can be concluded that the sum of the changes that are introduced by the inoculation procedure in a cast iron microstructure leads to an increase in its mechanical properties.

It should be noted that there is one more important indicator of the course of the inoculation: namely, the influence of primary austenite grains. However, this is most difficult to assess in practice. The authors in [8] described the basic issues that are related to increasing the number of dendrites in grey cast iron. The crystallisation of primary austenite in cast iron [9,10,11,12,13,14,15] is important due to the influence on the structure properties [16,17,18,19,20] and the appearance of such defects as porosity [21,22,23,24,25,26,27]. With increased dendrite dimensions, there is a high probability that the molten alloy will be enclosed in the interdendritic space. Consequently, a void is created in these areas as a result of the crystallisation, as well as the transition from the liquid phase to the solid phase; this creates the contraction porosity of the casting. Therefore, the first step in eliminating porosity in a casting is to develop a correct inoculation process for the primary crystallisation of the austenite in the cast iron; this will also affect the grains of the graphite eutectic. In summary, porosity is a common phenomenon in the production of cast iron. Its frequent cause may be a lowered sulphur content in the alloy (which should be 0.06–0.08% by mass in inoculated cast iron) [28].

Another reason for this is the increased nitrogen content in cast iron. The limiting solubility of nitrogen in grey cast iron is about 80 ppm (or 0.0080%). There are many sources of nitrogen in an iron foundry; one is a metal charge (especially steel scrap). Nitrogen may be present in carburisers and other alloying additives. Nitrogen can be absorbed from the atmosphere during the melting, and it can also be absorbed from the decomposition gases of some types of foundry sand, as well as the organic binders of moulds and cores. Very high nitrogen levels suggest the existence of multiple sources. It can be helpful to check the nitrogen level of the iron from several points during the process (raw material, molten reference alloy, inoculated alloy and from castings).

The purpose of the study is to investigate the method of the elimination of shrinkage porosity with the use of an inoculant increasing the nucleation of primary austenite grains in cast iron with a reduced sulphur content.

## 2. Methodology

### 2.1. Industrial Research 

In order to investigate the effect of the number of grains on the formation of porosity in grey cast iron, two castings were obtained in which defects such as porosity appeared. The castings were produced in a large foundry plant in Poland. The first example (A) was a casting of an electric motor shield that is used in methane mines. In the case of this type of casting, a lack of porosity is essential. The second example (B) was a lathe bed casting. Both castings were made of synthetic cast iron and possessed the chemical composition that is presented in Table 1. In both castings, porosity was detected in their structures.

The basic materials that are used for the production of grey cast iron are those that are shown in Table 2: that is, pig iron, steel and iron scrap, and alloy additives. The cheapest raw material that can be used for the production of cast iron is steel scrap. Cast iron that is obtained by carburizing steel is called synthetic iron.

The charge material was homogeneous steel scrap and iron scrap along with a graphite carburiser and ferroalloys. The charge was melted under industrial conditions in a six-ton (Mg) medium-frequency ABB induction furnace. After the melting, the molten alloy was kept at a temperature that was within a range of 1460 °C–1480 °C for 20 min. The chemical composition of the molten alloy was corrected with ferrosilicon Si75 and ferro-manganese FeMn80. Inoculants were used from one German producer (A) and one Norwegian producer (B).

**Figure 4:**
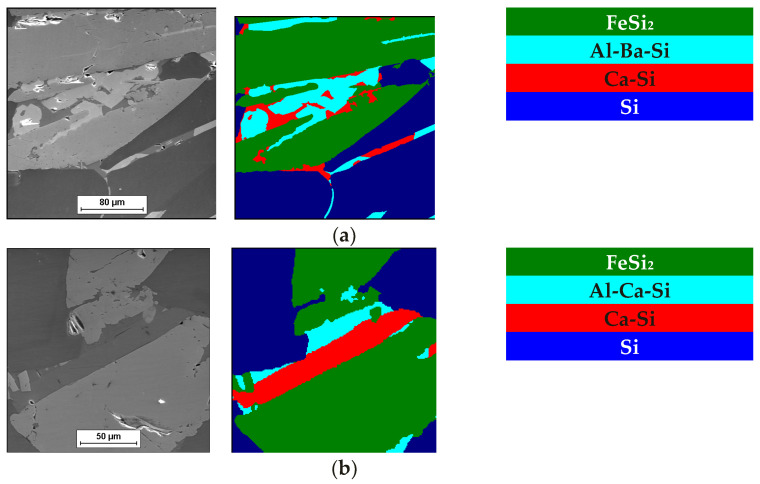
Distribution of main phases that were present in inoculants: (**a**) Type (A)—(FeSi_2_+Al-Ba-Si+Ca-Si+Si); (**b**) Type (B)—(FeSi_2_+Al-Ca-Si+Ca-Si+Si).

The chemical composition of the charge materials and additives that were used is summarised in Table 3, while the phase composition of the inoculants is shown in Figure 4. The images were made using the mapping technique that was carried out according to the methodology that was described in [29]. The phase regions that are shown in Figure 4 show that there were areas in the inoculant that affected the primary crystallisation of the austenite (e.g., Fe [8] Si, FeSi [30]) and the phases that influence the formation of the graphite nucleation sites, i.e., in this case, CaC_2_ and BaC_2_. BaC_2_ [3,4]. 

Both of the inoculants included Si, Ca, and Al. Inoculant (A) contained 1% Ba, and inoculant (B) had 0.25% more Si and Al.

**Table 2:** Chemical compositions of charge materials (% mass).

Metal Charge	C	Si	Mn	P	S
**Steel Scrap**	0.19	0.33	0.40	0.01	0.01
**Graphite Carburiser**	96.00	-	-	-	0.15
**Iron Scrap**	different chemical composition within current production				
**FeMn80**	0.50	1.00	80	0.25	0.03
**FeSi75**	0.15	75.00	0.50	0.05	0.04
**Silicon Metal**	-	>98.5	-	-	
**FeS**	-	0.3 max.	-	-	28–32

**Table 3:** Chemical compositions of inoculants (% mass).

Inoculants	Si	Ca	Al	Ba	Rest
**Inoculant (A)**	0.73	1.25	1.00	1.00	Fe
**Inoculant (B)**	0.73	1.5	1.25	-	Fe
**Fe Powder**	-	-	-	-	Fe

### 2.2. Laboratory Research

The tests were carried out in a medium-frequency induction furnace UFW 56-8, VEM Sachsenwerk (Dresden, Germany) with a crucible capacity of 15 kg (Mammut Type A-15)—Melt No. 3. The metal charges consisted of Sorel metal pig iron, steel scrap, technically pure silicon, ferromanganese, and iron sulphide. The chemical composition of the reference cast iron is presented in Table 4. The inoculation of the cast iron was carried out with the use of an Fe-Si inoculant (whose chemical composition is presented in Table 3) at an amount of 0.4% mass and iron powder at an amount of 0.2% mass.

The first chemical composition (Melt No. 1) refers to cast iron with a non-normative (reduced) sulphur content, while the second (Melt No. 2) refers to cast iron with a normative (favourable) sulphur content. The inoculation procedure was used for the reference cast iron. The melting procedure was as follows: after melting, the alloy was superheated to a temperature of 1490 °C and held at this temperature for about 120 s. The additional inoculants—iron powder—were introduced into the bath at a temperature of 1460 °C. The standard inoculants (A) and (B) were also introduced into the molten alloy when the temperature dropped to a value of 1430 °C. The cast iron microstructures were obtained from ⌀30 × 260 mm cast shafts.

### 2.3. Metallographic Analysis 

Samples were cut out, placed in mounting cups, and then poured over with acrylic resin. Then, using the Struers RotoPol-11 machine, they were ground using diamond discs 120, 220, 600, 1200 (rinsed with water) and polished on the MD-Nap disc with the use of diamond suspension and lubricant. The samples, after rinsing in ethyl alcohol and drying, were etched with Villela’s regent. 

The chemical analysis of the cast iron was performed using a HILGER spectrometer (Sterling, Margate, England). To evaluate the microstructure and porosity, a metallographic analysis was performed with a LEICA MEF-4M optical microscope supported by a LEICA-Qwin automatic image analyser, an “FEG-SEM” (field emission gun-scanning electron microscope) from Zeiss Merlin Compact, an INCA (Oxford) analyser, and the EDS model.

### 2.4. Thermal Analysis 

ITACA thermal analysis system stands for “incremental thermal and chemical analysis”. This system was developed by company ProService (Borgoricco PD, Italy) Technology. Heraeus Electro-Nite cups with a QC 4010 symbol were used for the analysis. These moulds were equipped with a K-type thermocouple and were free of any tellurium addition; as a result, the cast iron crystallisation took place in a stable system (cast iron crystallises as grey).

The ITACA [31] thermal analysis consisted of recording the crystallisation curve and determining its first derivative in real time. Based on the courses of these curves, the following parameters were determined:

- TLiquidus—liquidus temperature;
- TeStart—beginning of eutectic crystallisation;
- TeMin—minimum real temperature of graphite eutectic crystallisation;
- TeMax—maximum real temperature of graphite eutectic crystallisation;
- TSolidus—solidus temperature/end of eutectic crystallisation;
- REC—recalescence (determined as difference between TeMax and TeMin temperatures);
- PAE (eutectic austenitic precipitation)—this parameter is determined on the basis of the crystallisation time that elapsed from the beginning of the maximum real crystallisation temperature of graphite eutectic (TeMax) to the end of eutectic crystallisation (TSolidus);
- VPS—angle between the legs of the first derivative at solidus temperature.

### 2.5. Crystallization Simulation

The cast iron crystallization simulation was performed using the program ProCAST. Software (ProCAST 2016.1, ESI Group, Rungis, France), Visual-Environment14.5, ESI Group ®. Data used in the simulation are presented in Table 5. 

## 3. Assessment of Porosity

### 3.1. Analysis of Defects Arising in Casting (A)

**Figure 5:**
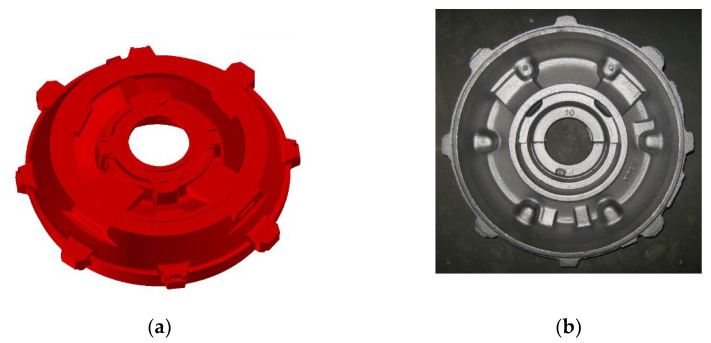
(**a**) Three-dimensional solid and (**b**) actual appearance of tested cast of electric motor shield.

**Figure 6:**
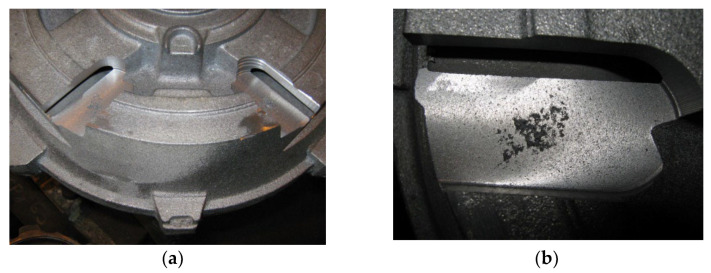
(**a**) Place of appearance of porosity in cast of motor shield and (**b**) appearance of defect.

One of the foundries struggled with the problem of obtaining a cast of an explosion-proof electric motor that was devoid of porosity defects. It seems that the resulting defect was of the shrinkage type, since the application of the inoculation procedure intensified the formation of this type of defect. The casting was made in a bentonite form on the LF-60.50 moulding line by Technical that was equipped with an FT-65A impulse moulding machine by Technical (Nowa Sól, Polska). Figure 5 shows the appearance of the 3D solid and a photograph of the engine disk cast. The chemical composition of the cast iron that was used to produce the target is presented in Table 1 and marked as Casting (A). Figure 6 shows the locations of these defects in the cast of the electric motor shield.

### 3.2. Analysis of Defects Arising in Casting (B)

**Figure 7:**
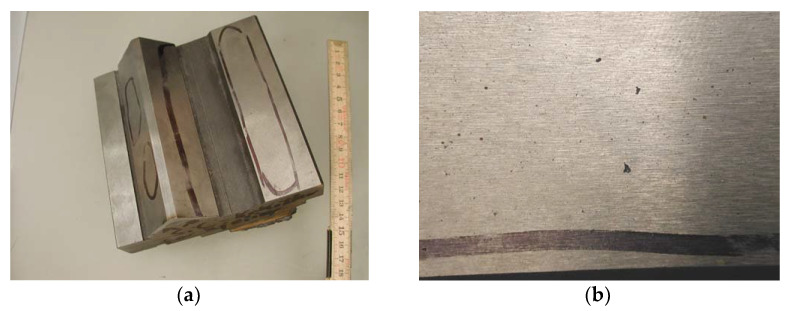
(**a**) Place of appearance of porosity in lathe bed casting and (unit: cm) (**b**) appearance of defect.

An examination of the lathe bed casting showed the presence of porosity. The casting was made in a furan form, which was filled with synthetic cast iron. A sample that was cut from the lathe bed is shown in Figure 7a. The sample showed voids (Figure 7b), which were revealed after machining.

**Figure 8:**
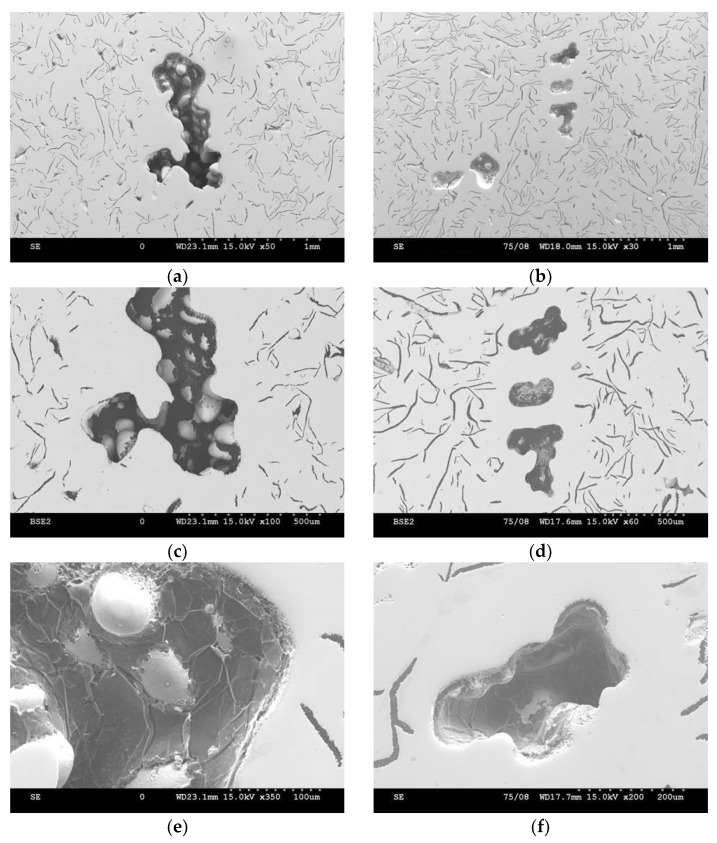
Revealed porosity on surface of lathe bed casting—single void at magnifications of (**a**) 50×, (**c**) 100×, and (**e**) 350×, as well as their grouping at magnifications of (**b**) 30×, (**d**) 60×, and (**f**) 200×.

**Figure 9:**
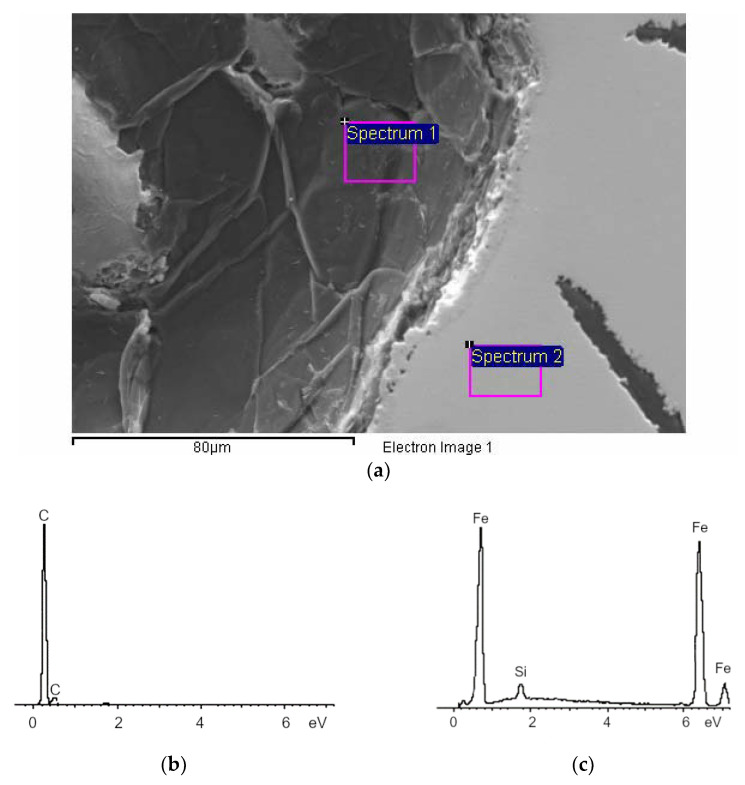
(**a**) Analysis of chemical composition of lathe bed casting on surface of internal porosity and metal matrix, (**b**) results of EDS analysis for inside of void, and (**c**) metal matrix.

The voids had irregular shapes, and one can see areas that were devoid of graphite at their boundaries (as shown in Figure 8). However, the interior of the voids was covered with graphite (shown in Figure 9). The irregular shapes of the pores is similar to a dendritic; this suggested a relationship with shrinkage during the crystallisation, which is typical of defects that appear with reduced sulphur contents in grey cast iron (although the nitrogen content may have exacerbated the problem). This type of defect is related to the crystallization of the molten metal confined in the interdendritic spaces.

## 4. Computer Modelling

**Figure 10:**
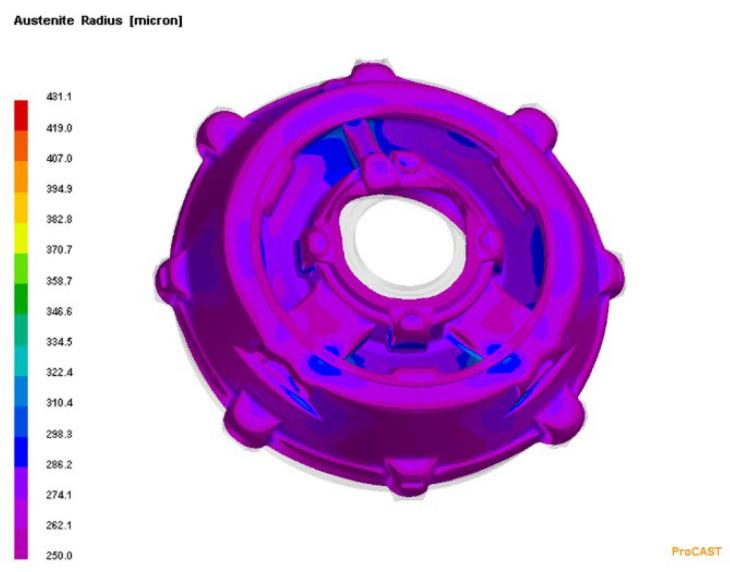
Distribution of primary austenite grains with diameters that were greater than 250 μm, with a small number of nucleation sites of primary austenite.

**Figure 11:**
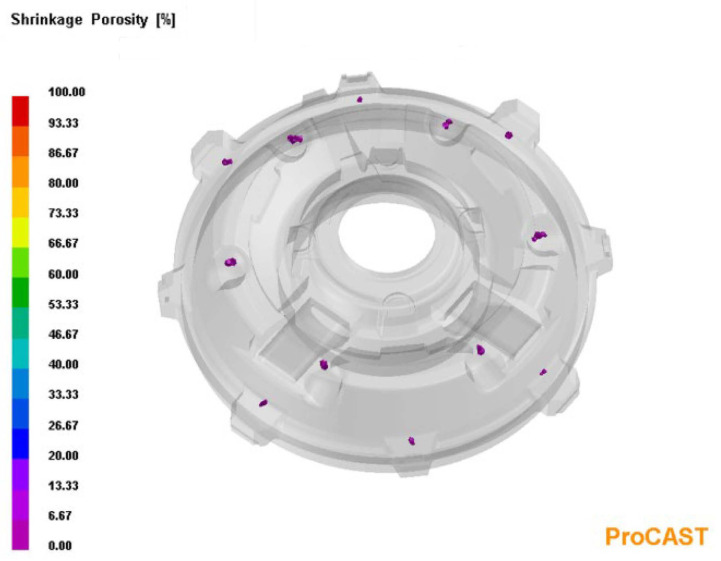
Distribution of microporosity in casting with small number of nucleation sites of primary austenite.

In order to visualise the mechanism of the microporosity formation in the casting that was related to the sizes of the primary austenite grains, the casting crystallisation was modelled in the PROCAST program. In this modelling, the parameter that corresponded to the average number of nucleation sites of the primary austenite grains changed. Reducing the number of austenite nucleation sites entailed an increase in the grain size. Figure 10 presents the modelling results that show the distribution of the primary austenite grains with diameters that were greater than 250 μm. Figure 11 presents distribution of microporosity in casting with small number of nucleation sites of primary austenite.

**Figure 12:**
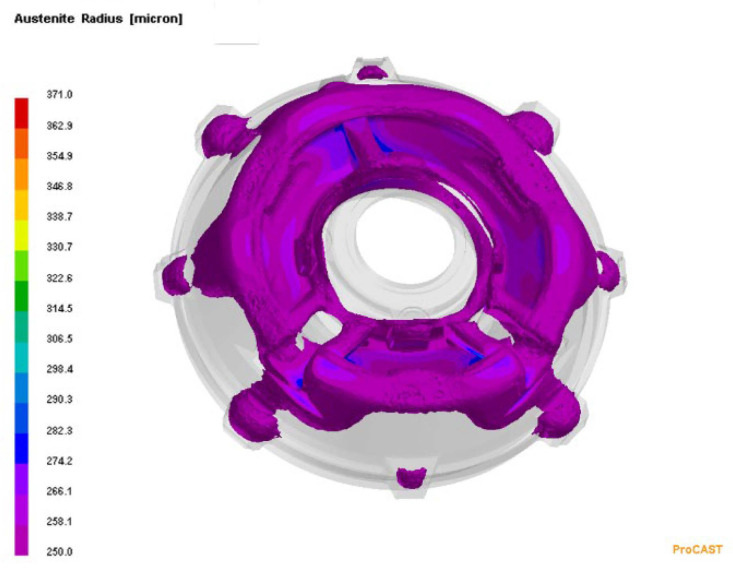
Distribution of primary austenite grains with diameters that were smaller than 250 μm, with a large number of primary austenite nucleation sites.

**Figure 13:**
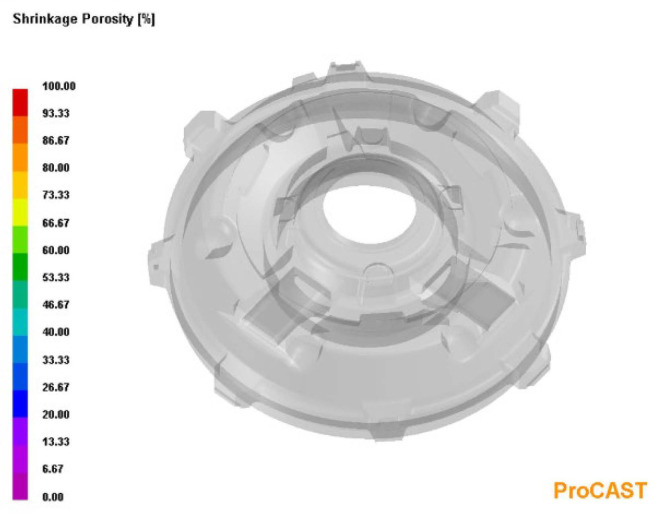
Distribution of microporosity in casting, with a large number of nucleation sites of primary austenite.

Figure 12 shows the modelling results that show the distribution of primary austenite grains with diameters that were less than 250 μm. The results of this modelling were obtained with the assumption of a large number of nucleation sites of the primary austenite grains. On the other hand, Figure 13 shows the lack of shrinkage porosity distribution for this case.

## 5. Laboratory Tests

**Figure 14:**
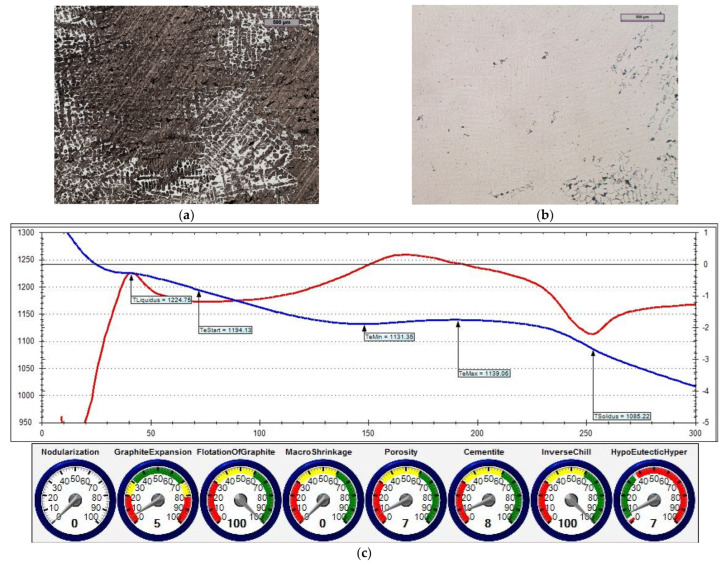
Reference grey cast iron (with 0.014% S content): (**a**) sample etched with Picral reagent; (**b**) non-etched sample; (**c**) changes in cast iron temperature (in time, sec.) during crystallisation and cooling of casting according to ITACA system.

The reference iron was melted with an unfavourable sulphur content (i.e., 0.014% S). Figure 14 shows the microstructure of the obtained cast iron as well as a screenshot that was obtained during the ITACA thermal analysis. 

**Figure 15:**
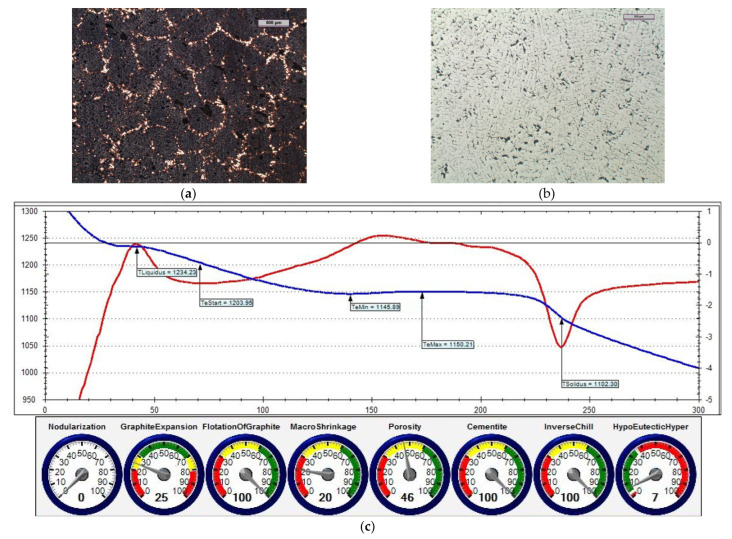
Grey cast iron (with 0.014% S content) and inoculated with 0.4% iron powder: (**a**) sample etched with Picral reagent; (**b**) non-etched sample; (**c**) changes in cast iron temperature (in time, sec.) during crystallisation and cooling of casting according to ITACA system.

In the first treatment, iron powder was added as an inoculant (at an amount of 0.4%) to reference cast iron. Figure 15 shows the microstructure of the cast iron that was obtained in this treatment, as well as a screenshot that was obtained during the ITACA thermal analysis. 

**Figure 16:**
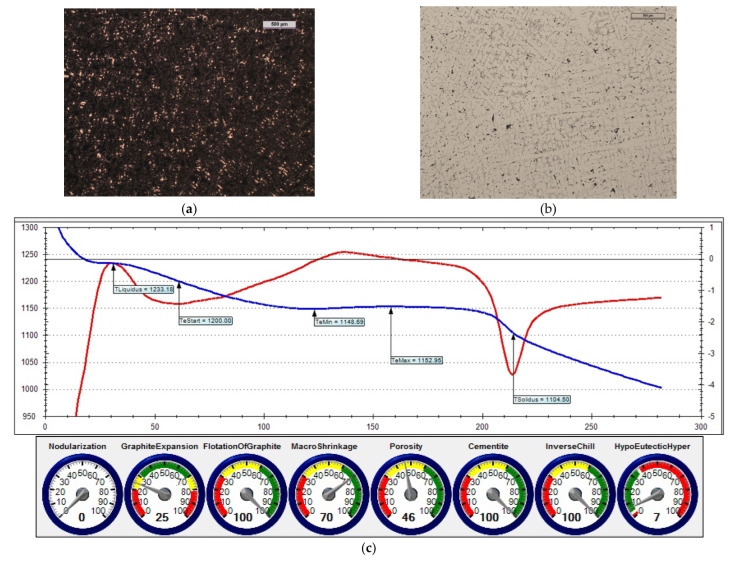
Grey cast iron (with 0.014% S content) with addition of 0.4% Inoculant (A): (**a**) sample etched with Picral reagent; (**b**) non-etched sample; (**c**) changes in cast iron temperature (in time, sec.) during crystallisation and cooling of casting according to ITACA system.

In the second treatment, the reference cast iron was inoculated by adding 0.4% of Inoculant (A) that was based on Fe-Si (which also contained Ca and Ba additives). Figure 16 shows the microstructure of the obtained cast iron, as well as a screenshot that was obtained during the ITACA thermal analysis. 

By assessing the POROSITY index (Figure 14c, Figure 15c and Figure 16c) that informed about the possibility of obtaining porosity in the grey cast iron casting, it can be concluded that the cast iron that was obtained thus far risked the appearance of porosity in the casting structure.

**Figure 17:**
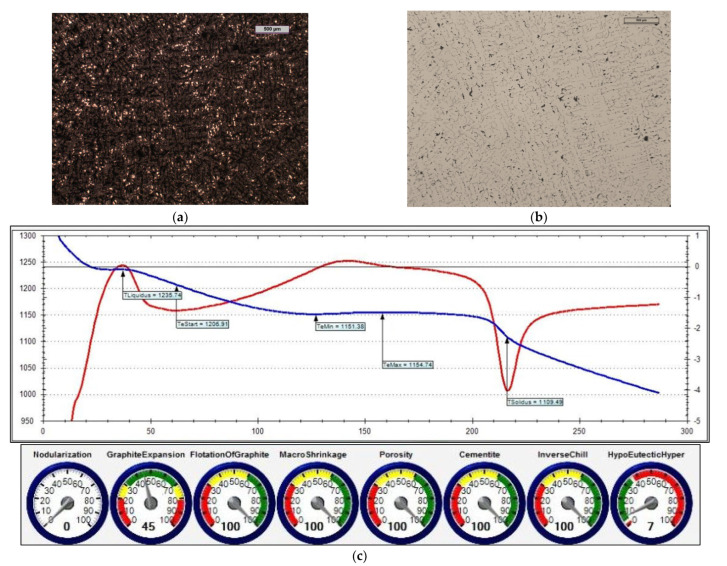
Grey cast iron (with 0.014% S content) with addition of 0.4% Inoculant (A) and iron powder (0.2%): (**a**) sample etched with Picral reagent; (**b**) non-etched sample; (**c**) changes in cast iron temperature (in time, sec.) during crystallisation and cooling of casting according to ITACA system.

In the third treatment, a physical mixture of Inoculant (A) (amounting to 0.4%) and iron powder (0.2%) was introduced into the reference cast iron. Figure 17 shows the microstructure of the obtained cast iron, as well as a screenshot that was obtained during the ITACA thermal analysis. 

**Figure 18:**
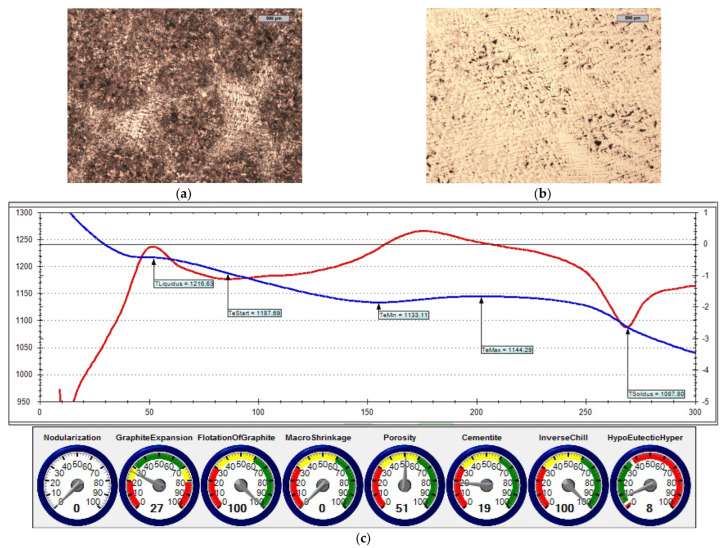
Reference grey cast iron (with 0.09% S content): (**a**) sample etched with Picral reagent; (**b**) non-etched sample; (**c**) changes in cast iron temperature during crystallisation and cooling of casting according to ITACA system.

**Figure 19:**
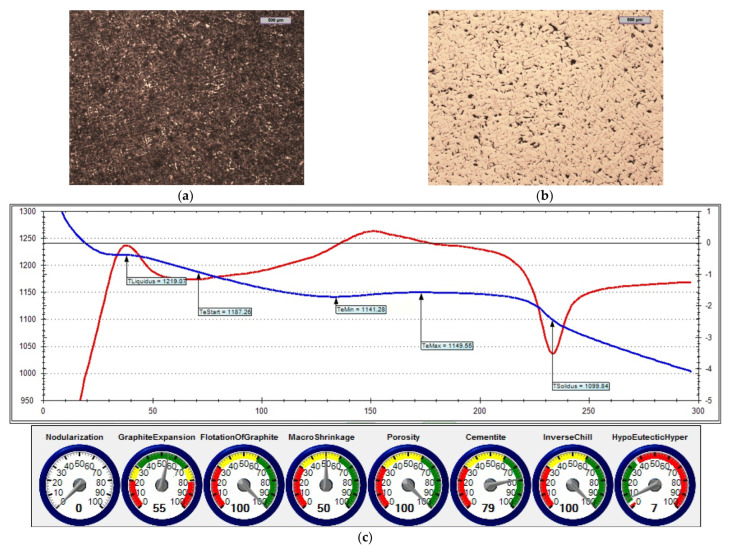
Grey cast iron (with 0.09% S content) with addition of 0.4% Inoculant (A): (**a**) sample etched with Picral reagent; (**b**) non-etched sample; (**c**) changes in cast iron temperature during crystallisation and cooling of casting according to ITACA system.

Table 6 summarises the crystallisation parameters for assessing the physicochemical state of the molten alloy according to the data that were measured by the ITACA system. As one can see, the ITACA thermal analysis is more practical to be used under the conditions of an industrial foundry. The very beginning of the crystallisation of the primary a-stenite dendrites for the liquidus temperature was clearly indicated in the graphic of the ITACA method (which has already been shown in Figure 14 and Figure 17). Table 7 additionally shows the parameters of the cast iron with the correct sulphur content; their graphic image is shown in Figure 18 and Figure 19. As can be seen in the graphic indicators from the ITACA analysis, the introduction of the inoculant with the Al-Ca-Si and Ca-Si phases to the reference cast iron with the increased sulphur content shows that it eliminated the phenomenon of porosity (Figure 4).

**Table 6:** Cast iron crystallisation parameters obtained in thermal analysis by ITACA method in Melt No. 1.

Melt No. 1	Chemical Composition of Cast Iron:							
	2.92% C, 1.65% Si, 0.38% Mn, 0.03% P, 0.014% S							
	ITACA thermal analysis parameters							
	T_liquidus_	Te_start_	Te_min_	Te_max_	Te_solidus_	Rec	VPS	PAE
Reference cast iron	1224.7	1194.1	1131.3	1139.0	1085.2	7.71	82	41
with 0.4% Fe	1234.2	1203.9	1145.9	1150.2	1102.3	4.32	28	53
with 0.4% Inoculant (A)	1233.2	1200.0	1148.7	1152.9	1104.5	4.25	23	47
with 0.4% Inoculant (A) and 0.2% Fe	1235.7	1206.9	1151.4	1154.7	1109.5	3.36	19	50

The second melting of the reference iron was carried out with the acceptable sulphur content (i.e., 0.09% S). Figure 18 shows the microstructure of the obtained cast iron, as well as a screenshot that was obtained during the ITACA thermal analysis. The reference cast iron was then inoculated with Inoculant B (Figure 19).

**Table 7:** Cast iron crystallisation parameters obtained in thermal analysis by ITACA method in Melt No. 2.

Melt No. 2	Chemical Composition of Cast Iron:							
	3.02% C, 1.61% Si, 0.40% Mn, 0.04% P, 0.09% S							
	ITACA thermal analysis parameters							
	T_liquidus_	Te_start_	Te_min_	Te_max_	Te_solidus_	Rec	VPS	PAE
Reference cast iron	1216.6	1187.7	1133.1	1144.3	1087.8	11.18	49	46
with 0.4% Inoculant (B)	1219.0	1187.3	1141.3	1149.5	1099.8	8.26	25	48

The list of parameters that was achieved in the ITACA thermal analysis system (presented in Table 6 and Table 7) shows that the simultaneous introduction of Inoculant A in an amount of 0.4%, together with iron powder in an amount of 0.2%, significantly changed the parameters of the physicochemical state that was responsible for the formation of microporosity in the casting. These parameters were as follows:

- TeMin—minimum real temperature of graphite eutectic crystallisation;
- VPS—angle between legs of first derivative at solidus temperature.

As related to the initial cast iron, the values of these parameters changed accordingly: TeMin (from 1131.3 to 1151.4) and VPS (from 82 to 19). The changes of these parameters directly translated into the "POROSITY" index in the ITACA thermal analysis.

## 6. Industrial Application

**Figure 20:**
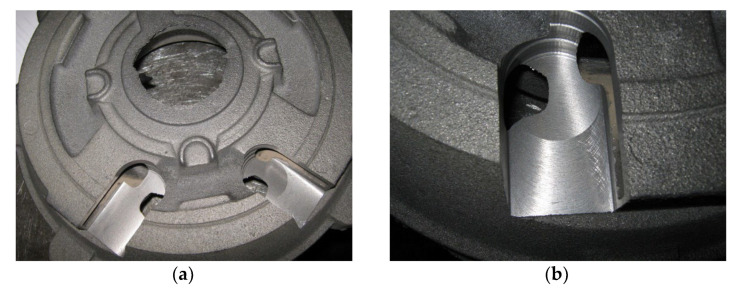
(**a**) Appearance of disc casting (no porosity-type defects) after applying special inoculation of cast iron with underestimated sulphur content of 0.02%, and (**b**) inoculated cast iron with sulphur content of 0.06%.

The research that is presented in this paper allowed for attempts to eliminate the porosity that forms in castings. For this purpose, an attempt was made to increase the number of primary austenite grains by introducing (before the actual treatment) a special inoculation of fine steel scrap mixed with steel shot (0.2 and 0.4% Inoculant [A] or Inoculant [B]) to molten metal. For a comparison, one melt was also carried out with a normal sulphur content. It should be remembered that the cast iron from which these castings were produced was synthetic cast iron, and its normal sulphur content is usually a maximum of 0.02%. Figure 20a shows a cast with an additional iron particle modification (IPM), and Figure 20b shows a cast with a sulphur content of 0.06% by mass. As one can see, introducing IPM and increasing the sulphur content to a normative level and then carrying out the inoculation allowed for the effective elimination of porosity defects in the casting. It should be emphasised that the supplementation of sulphur in synthetic cast iron creates problems in its dosing, in order to obtain the appropriate amount of S in the cast iron. The presented procedure also works well for eliminating porosity in a bed casting for a lathe.

## 7. Conclusions

The following conclusions can be drawn from the research results obtained:

- The results of the analysis in the research work show that the crystallisation of primary austenite grains is of key importance for the appearance of porosity in a casting made of grey cast iron with a low sulphur content.
- The defect shown indicates that it is shrinkage porosity.
- Choosing the right inoculants is the first and easiest step for eliminating these types of defects.
- The phase composition of the inoculant is essential to influence the primary (primary austenite grains) and secondary (eutectic grains-graphite + austenite grains) structure for cast iron.
- It is important to use a thermal analysis to analyse the metallurgical quality of the molten alloy. This allows for a quick assessment of whether the molten alloy will tend to crystallise with the formation of porosity-type defects.
- The use of a commercial inoculant containing FeSi_2_+Al-Ba-Si+Ca-Si+Si did not eliminate the porosity from the casting.
- The use of only iron powder also failed to produce a satisfactory effect of eliminating porosity in the casting.
- The preparation of a physical mixture and the application of an inoculant consisting of iron powder and a commercial inoculant FeSi_2_+Al-Ca-Si+Ca-Si+Si eliminated porosity defects in the tested castings.

## Figures and Tables

**Table 1:** Chemical composition of grey cast iron industrial research (% mass).

Melt No.	C	Si	Mn	P	S	Ti	Al	Cu	N
A	3.40	2.0	0.63	0.1	0.020	<0.01	-	0.8 Cu	-
B	3.24	1.60	0.91	0.04	0.025	<0.01	0.01 Al	-	0.013%

**Table 4:** Chemical composition of grey cast iron laboratory research (% mass).

Melt No.	C	Si	Mn	P	S
1	2.92	1.65	0.38	0.03	0.014
2	3.02	1.61	0.40	0.04	0.09

**Table 5:** Data used in the ProCAST simulation.

Linear Temperature Function of Temperature in the Liquidus–Solidus Range		
Heat transfer coefficient between mould and alloy, W/m^2^K	Liquid alloy	800
	Solid casting	400
Temperature, °C	Melt	1400.00
	Mould	20.00
	Liquidus	1165.55
	Solidus	1064.65
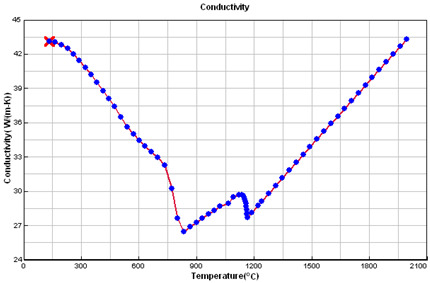	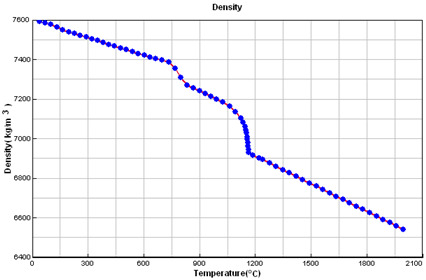	
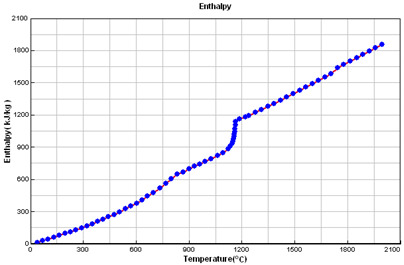	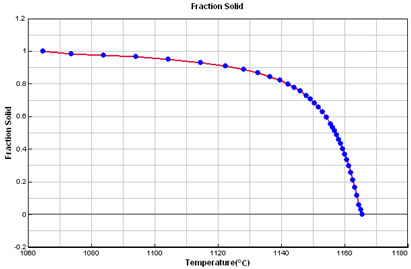	

## Data Availability

Not applicable.
